# Supplementary material for: Are carbon emissions trading and green financial instruments synergistic? -Comprehensive quantitative research based on content analysis
Source: PLoS One. 2024 Mar 7;19(3):e0298601. doi: 10.1371/journal.pone.0298601 (PMC10919601; doi:10.1371/journal.pone.0298601)
Supplement: S1 Data — (ZIP) [file pone.0298601.s001.zip › Data/地级市Z_F工作报告.zip!/╣π╬≈/┴°╓▌╩╨/└┤╫╘╣1⁄2╓┌║┼í╛┬φ┐╦╩2╛▌═°í┐_2017.docx]

各位代表：

　　现在，我代表市人民政府向大会作政府工作报告，请予审查，并请各位政协委员和列席会议的同志提出意见。

　　2016年工作回顾

　　过去一年，面对错综复杂的发展环境，我们在自治区党委、政府和市委的正确领导下，以五大发展理念为引领，深化供给侧结构性改革，做好稳工业、稳投资、稳消费、稳农业、稳进出口、稳财政“六稳”工作，大力开展实体经济发展年、重大项目建设年、精准脱贫推进年活动，突出创新驱动，加快产业转型、城市转型、社会转型，全力保持经济社会平稳健康发展，实现了“十三五”的良好开局。

　　——经济发展稳中有进。初步统计，全市实现地区生产总值2401亿元，增长7.2%；规模以上工业增加值1141亿元，增长6.5%；固定资产投资2338亿元，增长14%；财政收入370.16亿元，增长7.7%。经济发展呈现缓中趋稳、稳中向好的发展态势。

　　——质量效益稳步提升。三产比重提高2.7个百分点，对经济增长的贡献率达50%。规模以上工业企业利润121.7亿元，增长31%；高新技术企业突破200家，约占广西1/4；高新技术企业工业产值1456亿元，占规模以上工业总产值比重的1/3。单位生产总值能耗下降4.5%。非税收入占一般公共预算收入的28%，占比降低0.6个百分点。经济发展的协调性进一步增强。

　　——生态环境持续改善。投入生态环保园林建设资金4.6亿元，增长32.4%。市区空气质量优良天数316天，优良率为86.3%，提高了3.1个百分点；细颗粒物浓度均值（PM2.5）下降10.7%、可吸入颗粒物浓度均值（PM10）下降4.8%；柳江河饮用水保护河段保持国家地表水Ⅲ类以上水质标准。全市森林覆盖率达65.03%，城市建成区绿化覆盖率达43.25%，城市人居环境持续改善。

　　——人民生活获得更多实惠。全市财政民生支出270.2亿元，增长8.3%，连续五年保持增长，占一般公共预算支出的79.6%。城镇居民、农村居民人均可支配收入分别为30733元、10347元，分别增长7%、9.5%。城镇登记失业率3.95%，控制在自治区下达指标以内。就业稳、物价稳，人民群众的获得感不断提升。

　　一年来，我们做了以下工作：

　　全力以赴做好稳增长工作。面对持续加大的经济下行压力，我们把稳增长作为政府工作的首要任务，出台加强要素保障、减轻企业成本41条措施，实施供给侧结构性改革总体方案及系列政策，推动“三去一降一补”得到有效落实。在降低制度性交易、企业融资、用地等方面为企业降低各类成本超过15亿元，实施“营改增”试点为企业减税9.25亿元，落实各项税收优惠累计为企业减免税款28.7亿元；实行北环高速柳州车辆免费通车，降低企业物流成本；推出购房补贴、购房券等政策鼓励新市民租房、购房，柳州成为广西首个公租房货币化试点城市。突出抓好经济运行、企业帮扶、政策落实、环境优化，经济增长保持在合理区间，稳住了经济发展的基本面。柳州获得国务院稳增长表彰，成为国家小微企业创业创新基地城市示范。

　　大力推进产业结构调整。工业结构不断优化。实施“十大工业结构调整工程”“工业八大专项行动”，预计完成全部工业总产值4742亿元，增长4%。汽车工业继续领跑全市产业，汽车产量突破240万辆，增长5.6%；上汽通用五菱宝骏基地二期、新能源汽车生产基地一期和东风柳汽柳东乘用车基地二期建成投产，多款新能源汽车、“T动力”乘用车等车型相继问世，上汽通用五菱成为全区首家销售收入突破千亿元企业。新型钢材比重持续上升，柳钢钢材产量突破1400万吨，实现扭亏为盈。机械工业加快向先进装备制造升级，主流产品实现更新换代，累计产值增速实现正增长。战略性新兴产业占全市工业比重达11%。自主创新能力持续提升，新认定自治区级工程技术研究中心5家、自治区级企业技术中心8家，

　　东风柳汽成为国家技术创新示范企业；两化融合不断深化，柳钢集团等6家企业成为国家两化融合管理体系贯标试点企业。质量强市战略深入实施，东风柳汽、广西建工五建公司获第三届 “自治区主席质量奖”，历届全市获奖企业累计有5家，占广西近半壁江山。园区经济总量占全市比重68%，柳东新区成为国家小微企业创业创新示范基地，阳和工业新区成为广西首个国家产业集群区域品牌建设试点。现代服务业快速发展。预计全市社会消费品零售总额1038.5亿元，增长10.0%；实现第三产业增加值931.7亿元，增长10.1%。柳南万达建成开业，华润、恒大、毅德等商业综合体项目进展顺利，钢铁、农贸等千亩级物流园运营良好。“电商柳州”加快推进，跨境电商产业园建成并投入运营，阿里巴巴·柳州产业带入驻企业1000家，鹿寨县、融安县成为“国家电子商务进农村综合示范县”，全市电子商务交易额达605.1亿元，增长36%。螺蛳粉产业园建成开园，方便装螺蛳粉销售收入超15亿元。房地产市场健康平稳，库存消化周期为3.5个月。旅游名城建设深入推进，百里柳江创建5A级景区，全国首趟旅游扶贫始发动车——“柳州三江号”成功开行，三江县成为国家旅游标准化示范县。全市旅游总收入356.7亿元，增长25%。现代农业稳定发展。现代农业示范建设取得新成果，3个自治区级、5个市级、13个县级、15个乡级现代农业示范区通过自治区验收；粮食总产量稳定在85万吨左右，水果、桑蚕、茶叶、蔬菜、水产畜牧养殖等特色产业规模生产，11万亩“双高”糖料蔗基地稳步推进，成功举办首届全国甘蔗机械化博览会。“农村电商”工程建成100个“农村淘宝”村级服务站。注册登记农民专业合作社由1861家增至2203家，农业产业化重点龙头企业由102家增至110家。全市农林牧渔业总产值296.8亿元，增长3.7%。

　　稳步开展精准脱贫工作。安排财政专项扶贫资金9.15亿元，整合各部门资金27.17亿元，实施脱贫攻坚“七个一批”“十大行动”，完成精准识别建档立卡，实现脱贫8万人，脱贫摘帽贫困村60个，圆满完成自治区下达的年度目标任务。开展基础设施建设大会战，建成建制村硬化路48条534.8公里，修建屯级道路597条1155公里，解决1.5万贫困人口饮水问题。实施精准产业扶贫，扶持贫困户发展果蔬、桑蚕、禽畜养殖等产业，受益建档立卡贫困户3.1万户15.2万人。开展扶贫小额信贷工作，为1.83万户发放扶贫小额信贷8.8亿元。推进32个易地扶贫搬迁项目，落实建设用地151公顷，实施扶贫搬迁21152人。结对帮扶、社会帮扶、教育扶贫、健康扶贫等工作扎实开展。

　　进一步加快城乡建设步伐。国家新型城镇化综合改革试点扎实开展，寨沙和沙塘全国建制镇示范试点等工作稳步推进。科学编制柳州市近期建设规划（2016—2020年），全面启动新一轮城市总体规划编制，“多规合一”试点扎实开展。城市“一主三新”加快布局，柳东新区成为国家产城融合示范区，柳江撤县设区正式挂牌，北部生态新区启动建设。城市承载功能进一步提升，城市轨道交通前期工作进展顺利，柳州站站房扩建工程（西站房）基本完成，雒容铁路商品汽车发运基地投入使用，桂柳高速完成路面改造，三江至柳州高速公路基本建成，柳东大道延长线全线贯通，红花二线船闸开工建设，柳州机场新航站楼竣工使用，古偿河水库大坝主体完工，落久水利枢纽等重大项目加快推进。城市基础设施不断完善，白沙大桥、西外环等项目进展顺利，智慧城市、海绵城市和地下综合管廊试点建设有序开展。生态建设取得新进展，“美丽柳州·生态乡村”深入推进，村屯绿化、饮水净化等工作取得明显成效；花园城市2.0版启动建设，环江滨水大道展现新景观。

　　全面深化改革、扩大开放。 “八个一”综合改革深入推进，“一枚印章管审批”实现动态管理、运行正常；“一份清单管边界”由41个市直部门向社会公布权力清单、责任清单。深化国有企业改革，组建了轨道交通等九大集团公司。商事制度改革扎实推进，全市新登记注册市场主体3.79万户，增长7.6%。金融改革不断深化，87个政府性投资项目融资到位761亿元，融资总量再创历史新高；建立PPP项目储备库，引入PPP项目社会资本80亿元；银行业金融机构本外币各项存贷款余额为3350亿元、2300亿元，分别增长18.68%和12.12%；新增4家企业在“新三板”挂牌上市，率先在全区实现企业“新三板”股权质押融资。农村产权制度改革以及财税、科技、文化、医疗、教育等各项改革稳步推进。积极融入国家“一带一路”、珠江—西江经济带开放开发，粤桂黔高铁经济带合作试验区广西园落地建设，成功举办第二届粤桂黔高铁经济带合作联席会议、第六届中国—东盟汽车工业博览会。招商引资成果丰硕，引进市外境内到位资金848.23亿元，增长14.03%；实际利用外资（全口径）5.98亿美元。

　　加大民生保障力度。为民办实事项目全面完成。“大众创业、万众创新”扎实开展，城镇新增就业6万人，农村劳动力转移就业6.1万人。社会保障制度实现城乡居民全覆盖，五项社会保险参保人数378.3万人，连续十二年上调基本养老标准；市区城市低保标准提高到每人每月500元，市区农村低保标准提高到每人每年3200元。棚户区实物建房建成14767套、分配入住11000套，农村危房改造新开工15304户、竣工11365户。义务教育均衡发展取得新成果，县属轮教、集团化办学效果良好，市区义务教育学校基本建设“三年攻坚”任务基本完成，新增学位5.52万个，全市中小学教职工编制增加20.7%。普通高中办学水平和教育质量不断提高。全国首批现代学徒制试点工作顺利推进。职业教育国际化、高等教育加快发展。文体事业欣欣向荣，舞蹈诗《侗》《八桂大歌》等精品艺术影响力不断扩大，“鱼峰歌圩”成为国家公共文化服务示范项目。华侨城文化旅游科技体验园等项目扎实推进。东门等历史街区保护整治顺利完成，成功举办2016国际水上狂欢节、第九届奇石节，全民健身活动丰富多彩。卫生计生事业不断进步，市、县公立医院改革稳步实施，区域医疗联合体建设、远程医疗服务扎实开展，中医药服务能力、疾病防控工作水平得到提升。依法稳妥实施全面两孩政策，计划生育服务管理转型升级步伐加快。深入推进“平安柳州”建设，改造升级“天网”工程，社会治安防控体系进一步完善。安全生产形势持续稳定好转，食品药品监管不断加强，依法调处化解各类纠纷，保持社会和谐稳定。国防教育深入开展，全民国防意识不断增强，国防动员准备和后备力量建设扎实推进，成功召开全区民兵应急分队规范化建设现场会，形成“柳州经验”、打造“柳州模式”，实现全国双拥模范城“八连冠”。

　　不断加强政府建设。扎实开展“两学一做”学习教育，学习贯彻落实党的十八届五中、六中全会精神，狠抓政府作风转变。加大法治政府建设，启动实施“七五”普法，柳州首部实体法——《柳州市莲花山保护条例》获自治区人大常委会批准，首部程序性政府规章——《柳州市政府规章制定程序办法》正式施行并经国务院法制办备案。认真执行人大决议决定，主动接受人大及其常委会法律监督、工作监督和政协民主监督，市人大代表议案建议、市政协委员提案办结率和满意率均达100%。坚持 “市长接待日”“市长企业接待日”制度，开展“中小企业服务月”活动，积极为企业、群众排忧解难。认真贯彻中央“八项规定”精神和国务院“约法三章”，加强行政监察、审计监督，严厉查处各类违纪违规违法行为。圆满完成市县乡三级政府换届，确保了换届风清气正。

　　过去一年，全市人事、编制、外事、侨务、口岸、税务、物价、统计、国家安全、决策咨询、档案、地方志、气象、水库移民、供销、民族宗教、老龄、妇女儿童、残疾人事业、防汛抗旱、防震减灾、社会科学等各项工作都取得新的成绩，作出了新的贡献。

　　在总结成绩的同时，我们也清醒地看到，全市经济社会发展中还存在不少困难和问题：稳增长、调结构压力很大，既要面对新常态下经济下行压力，又要面对经济转型带来的各种阵痛；发展的短板问题依然突出，县域经济实力不强，非公经济不旺，开放型经济发展不快；影响社会和谐稳定的矛盾和问题还比较多，信访维稳等问题不容忽视，脱贫攻坚任务重、难度大、时间紧，群众就业、就学、出行和住房等困难仍然存在。对于这些问题，我们将高度重视，认真研究，采取有力措施，切实加以解决。

　　各位代表，过去一年取得的成绩，是自治区党委、政府和市委正确领导的结果，得益于市人大、市政协的监督和支持，凝聚了全市各族人民的辛勤与智慧。在此，我代表市人民政府，向全市各族人民，向驻柳人民解放军、武警驻柳部队官兵，向各民主党派、工商联、人民团体和海内外所有关心、支持柳州发展的各界人士，致以崇高的敬意和衷心的感谢！

　　2017年工作总体要求与目标

　　今年，是实施“十三五”规划的重要一年，是供给侧结构性改革的深化之年。做好今年的工作，机遇与挑战并存，我们将认真贯彻落实中央、全区经济工作会议精神，按照郑俊康书记在中共柳州市第十二届代表大会第二次会议工作报告中提出的总体要求、目标任务和工作部署，坚定发展信心决心，做好克难攻坚准备，始终保持想干事、敢干事、干成事的精神状态，不断开拓发展新境界，推动柳州经济社会发展迈上新台阶。

　　今年政府工作的总体要求是：全面贯彻党中央、国务院和自治区党委、政府的决策部署，继续按照稳中求进工作总基调，坚持以提高发展质量和效益为中心，以推进供给侧结构性改革为主线，秉持五大发展理念，实施全面创新、城乡一体、绿色崛起、开放带动、共享发展战略，深入推进“实业兴市，开放强柳”，着力营造风清气正的政治生态、团结和谐的社会生态、山清水秀的自然生态，全力打好产业转型升级、基础设施建设和精准脱贫攻坚战，力争在实体经济发展、项目投资建设、保障改善民生、全面深化改革取得新突破，为开创“十三五”发展新局面、实现“率先建成”和“打造龙头”双目标打下坚实基础。

　　2017年柳州经济社会发展的主要预期目标是：地区生产总值增长7.5%左右，在实际工作中争取更好结果；规模以上工业增加值增长6.5%，固定资产投资增长14%，财政收入增长7%，社会消费品零售总额增长10%，城镇居民人均可支配收入增长8.5%，农村居民人均可支配收入增长10%。城镇登记失业率和单位生产总值能耗、主要污染物排放总量控制在自治区下达指标以内。

　　在今年的政府工作中要重点把握好以下几点：一是牢牢把握稳中求进工作总基调。立足于“稳”、着眼于“进”，坚持实业主导、投资引领、生态优先、民生为重、全面发展，走质量高、效益好、结构优、可持续的发展之路。二是大力振兴实体经济。柳州振兴实体经济的主战场在制造业，关键在存量，突破在增量，动力在创新。通过提升创新能力，推动传统产业提质增效，加快发展战略性新兴产业，建设智能制造产业新基地，加快新旧动能顺利转换。三是全力推进供给侧结构性改革、扩大对外开放。抓住供给侧结构性改革这条主线，深入推进“三去一降一补”，保障原有政策落地生效的同时，根据新情况新问题完善政策措施，不断提高供给体系的质量和效率。大力实施开放发展战略，主动融入广西“四维支撑、四沿联动”开放发展新格局，增强柳州在区域经济中的辐射力、带动力，加快建设成为内陆开放型经济新高地。四是不断提高城市建设管理水平。以北部生态新区、柳江新城区建设拓展城市发展的新空间，以轨道交通为引线构建城市发展的新格局。坚持“一江两岸”生态保护优先、适度开发，打造具有柳州特色的新型城镇化品牌。牢牢抓住影响城市安全、阻碍城市发展、群众反映强烈的突出问题，持续用力补好短板，提升城市综合竞争力。五是更加重视保障和改善民生。把脱贫攻坚作为第一民生工程来抓，坚决打赢精准脱贫攻坚战。优先保障民生支出，加强义务教育、医疗卫生、社会保障、公共文化、环境保护等基本公共服务，更加关注贫困群众、弱势群体的生活，让人民群众在共建共享中享有更多获得感。

　　2017年工作安排

　　围绕今年的目标任务，我们将重点抓好以下几个方面工作：

　　一、实施创新驱动发展战略，打好产业转型升级攻坚战

　　优化提升传统产业。加快支柱产业结构调整优化，汽车工业完善全产业链布局，竣工上汽通用五菱柳州发动机五期工程、东风柳汽柳东研发中心等项目；实施汽车零部件再升级改造计划，通过主机厂主导、外部技术整合、系统创新创造，提高制造水平和本地配套率。钢铁产业着力调整产品结构，开发冷轧汽车用钢，推动柳钢产品向高附加值、高品质、高科技转型。机械产业支持工程机械、建筑机械优化升级，拓展现代农业机械、机器人两大新产业板块。依靠科技创新、技术更新改造，提升化工、纺织服装、建材等传统产业和以螺蛳粉为代表的食品加工产业竞争力。

　　培育壮大战略性新兴产业。对接“十三五”国家战略性新兴产业发展规划和“中国制造2025”，加快构建战略性新兴产业体系。大力发展新能源汽车，建设上汽通用五菱年产20万辆新能源车、东风柳汽新能源汽车等项目，支持新能源汽车核心零部件研发生产，建设一批充电桩等配套设施，构建较为完整的新能源汽车生产体系。大力发展智能制造产业，与中国南方电网合作建设智能电网产业园，抢占南方电网在西南地区布局制造智能电网器件的制高点。以“项目+产业”模式，全力引进中车股份等城市轨道交通装备制造企业，合作建设集整车生产、保养、维修为一体的维保中心，吸纳聚集一批轨道交通零部件生产企业，推动城市轨道交通装备产业化发展。以柳州列为自治区建筑产业化试点示范城市为契机，推进柳钢与中建钢构合作建设生产基地、与建工集团合作生产建筑标准件，发展节能材料、新型建筑模块、住宅标准件产业，促进钢铁、铝材、水泥、工程机械与建筑产业深度融合。建设战略性新兴产业示范基地，重点培育30户战略性新兴产业骨干企业，加快将新能源汽车、新一代信息技术、智能装备制造、新材料、节能环保、生物医药等战略性新兴产业培育成为先导性、支柱性产业。

　　增强创新驱动发展能力。发展工业设计产业，在北部生态新区规划建设“工业设计城”，大力引进国内外工业设计企业和设计研发机构。推进公共创新平台建设，支持大中型骨干企业建立企业技术中心，培育一批自治区级、市级企业技术中心，加快国家级汽车质量监督检验中心、柳州新能源汽车检测平台等项目建设。深入推进国家小微企业创业创新城市示范工作，建设柳东新区、阳和新区“智能制造”“云服务平台”等示范基地，新增30家国家高新技术企业、30个市级以上众创空间、30家市级以上小微企业创业创新基地。发扬“工匠精神”，深入实施质量强市战略，争取率先建成广西首个“全国质量强市示范城市”。

　　提升产业集聚化发展水平。大力推进北部生态新区建设，引导生态工业、智能制造、现代服务业向北部生态新区集聚。全面推进柳东新区（广西柳州汽车城）先进制造业核心产业区，以汽车整车生产为龙头，促进汽车零部件、电子信息等产业集聚发展。落实园区工业用地价格、厂房租赁补贴等优惠政策，大力推进产业项目建设。加快完善阳和工业新区、河西工业园、柳北工业园、洛维工业园、新兴工业园等园区基础设施，不断增强园区综合承载力。促进各县工业集中区集约化、差异化发展，打造特色产业基地。

　　二、提高农业现代化发展水平，统筹推进城乡协调发展

　　加快城乡一体化进程。建设柳江新城区，推进鹿寨、柳城同城化发展。以柳东大道延长线贯通为契机，促进中心城区、柳东新区与鹿寨产业优势互补、错位发展。发挥三柳高速公路交通动脉作用，着力将柳城、融安、融水、三江四县打造成为国家重点生态功能区、农产品主产区、生态民俗文化旅游带。开展白莲机场至三江市域城际铁路前期工作，开工建设桂林至柳城高速公路，优化城乡发展布局，促进城乡资源共享。

　　大力发展现代农业。集中力量打造一批特色农业示范区，创建2个以上自治区级农业示范区，争创国家级现代特色农业示范区。调优农业产业结构，提高粮食生产保障能力，发展水果、桑蚕、茶叶等优势特色产业，推动柑橘产业健康发展，着力打造国内高品质柑桔生产基地、优质金桔生产基地。加快 “双高”糖料蔗基地建设。发展水产畜牧生态健康养殖，构建农牧结合、种养加一体的现代农业产业体系。推进现代农机制造，提高农业机械化水平。规划建设农产品精深加工园区，培育一批粮食烘储加工、果蔬茶加工、农产品冷链物流企业。实施农产品质量安全战略，创建农产品质量安全县，构建农产品质量安全追溯及监管体系。

　　完善农业基础设施。市本级财政安排6.65亿元支持各县发展。实施“县县通高速、村村通公路”工程，确保全市具备条件的建制村通畅率达到100%，开工建设200公里县乡联网公路。启动第三批整县推进高标准基本农田土地整治，实施农村人饮工程，解决4万农村人口饮水困难问题，新增恢复改善农田灌溉面积60万亩。推进落久水利枢纽主坝、副坝建设，做好移民搬迁安置，力争开工建设洋溪（梅林）水利枢纽。统筹实施农村电力沼气、宽带通讯、垃圾处理等建设，不断改善农村生产生活条件。做好传统村落、少数民族特色村寨保护开发，建设特色名镇名村。

　　增强县域经济发展活力。做好柳江由县到区过渡期内的建设，综合开发利用土地资源，增强产业经济活力。推进鹿寨县撤县设区工作，优化发展汽配、化工、能源、林木加工等产业。柳城县发展汽车零部件、制糖、机械制造、新材料、生物制药等产业，促进产业向园区集聚。融安县发展制药、香杉、竹木加工业、风电水电产业，打造金桔特色产业，建设绿色低碳生态工业基地。融水县做大苗乡特色生态旅游，发展高山茶叶、中草药、林下种养等生态产业。三江县提升茶叶质量与品质，发展稻田养鱼等特色产业，努力建设成为全国少数民族特色旅游目的地。

　　三、大力发展现代服务业，促进第三产业优质高效发展

　　加快打造广西生产性服务业新高地。实施汽车产业、现代物流、工业设计创新服务工程，大力发展汽车后市场产业，建设汽车后市场产业园，进一步增强服务业的聚集力。围绕城市快环经济圈，布局建设一批区域性大型商品交易市场，推进西鹅铁路货运中心、毅德商贸物流城等项目建设。发展研发设计、信息技术服务等产业，推动生产性服务业向专业化和价值链高端延伸。

　　提升生活性服务业品质。推动生活性服务业便利化、精细化、品质化发展。进一步繁荣城市商圈，加快河北半岛商圈特色发展，完善河东文昌商圈功能布局，规划建设城市生活用品物流中心、冷链物流产业园。推进恒大、华侨城文化旅游科技体验园、新柳邕（二期）等项目建设，引导传统商贸业转型升级。坚持“房子是用来住的、不是用来炒的”的定位，合理增加土地供应，加强住房市场监管，规范开发、销售、中介等行为。积极创建百里柳江、程阳八寨5A级景区，支持三江、融水、鹿寨创建广西特色旅游名县，推动融水“国家全域旅游示范区”建设，打造鹿寨县中渡镇国家级特色小镇。

　　发展现代互联网产业体系。实施“互联网+”行动计划，鼓励骨干企业开放创新交互平台，支持柳钢集团电商平台、东风柳汽车辆增值服务平台建设。实施“电商柳州”工程，发展工业电商、供应链电商、大宗商品电商，实现线上服务资源与线下产业资源深度融合。加快柳城县、鹿寨县、融安县“电子商务进农村示范县”建设，推进“互联网+”与农业、旅游等行业融合，实施阿里巴巴“农村淘宝”项目，活跃农村电子商务。

　　建设区域性产业金融服务中心。以服务实体经济为导向，建立政策性融资担保体系，由政府出资组建政府性融资担保机构，解决中小微企业融资担保问题。围绕产业发展需求，通过政府出资并吸纳金融机构和社会资金，设立产业发展投资基金，引入风险投资机制，扶持新兴技术企业、高科技企业发展。与有资质的金融机构合作建立产权、股权交易平台和金融产品交易平台，拓展企业融资的新模式。发展多层次资本市场，支持金融投资集团取得基金、信托、保险等金融牌照，积极防范区域性金融风险。

　　四、全力打好精准脱贫攻坚战，确保8.5万贫困群众脱贫

　　全面推进脱贫攻坚。深入实施精准脱贫“七个一批” “十大行动”，建设脱贫攻坚大数据管理平台，扎实推进特色种养、移民搬迁、旅游发展、就业扶持、医疗救助、教育帮扶、低保兜底等脱贫攻坚工程，提高脱贫工作的精准性和有效性，确保年底融安县脱贫摘帽，全市85个贫困村脱贫出列，减少贫困人口8.5万人。

　　加快补齐基础设施建设短板。推进贫困村屯级道路、农村电网、宽带网络等建设，实现贫困地区20户以上自然屯通屯硬化道路800公里；完成4658户贫困户危房改造；对200个贫困村（屯）老旧供电网络实施更新改造，建设一批高效节能配电台区；推动光缆入村入屯入户，年内基本实现贫困村宽带信息网络全覆盖；按照“六个一”标准，建设45个贫困村公共服务中心。

　　进一步增强脱贫实效。严格“一把手”脱贫攻坚责任制，实行督查、通报、协调制度，抓好扶贫开发成效考核和实绩考核。加强农村低保制度与扶贫开发政策衔接，制定农村低保标准动态调整方案，完善农村低保标准与物价上涨挂钩联动机制，推广孤寡贫困户“集中安置+供养”模式。进一步完善精准脱贫工作机制，抓好群众动员工作，全面推进“百企扶百村”活动，广泛动员社会力量共同参与扶贫。

　　五、坚持以项目投资为抓手，打好基础设施建设攻坚战

　　掀起新一轮城市建设新高潮。大力实施“十三五”期“十大城市建设工程”，推进城市轨道交通、柳东新区文化广场、市民服务中心等项目建设，加快实施五岔路口改造、官塘大桥、白沙大桥等项目，进一步完善城市基础设施体系。为迎接自治区成立60周年大庆，全面启动具有公益性、标志性的60个重大项目建设，并争取2018年底前基本建成或实现开工。全力实施年度1045个城建项目，计划投资555.2亿元。

　　全力扩大有效投资。集中力量实施503项市级层面重大项目，年度计划投资763.2亿元，其中：产业发展投资231.7亿元，民生保障投资176.3亿元，生态环保和节能减排投资45.6亿元。着力抓好工业投资，实施新一轮技术改造工程，制定促进工业投资考核激励办法，集中资金扶持重大工业项目投资、中小企业融资担保和重大工业项目前期工作。围绕智能制造、轨道交通产业化、建筑产业化等领域，超前谋划、储备一批战略性项目，不断增强投资后劲。

　　强化项目建设要素保障。实施重点项目推进机制，倒排工期、挂图作战，推动项目建设提速提效。继续开展打击“两违”专项行动，依法开展征地拆迁，全力保障重大项目用地需求。继续加大重大项目建设筹融资力度，拓宽项目建设融资渠道，推广政府和社会资本（PPP）合作模式，力争年内政府和社会资本合作项目投资达100亿元。加强与各类金融机构合作，充分发挥各类投资基金的撬动作用。

　　六、推动各项改革举措落地生效，增创体制机制新优势

　　深化经济领域重点改革。狠抓供给侧结构性改革“1+5”政策文件落实，推动“三去一降一补”工作任务取得更大进展。深化国资国企改革，推进国有企业重组整合，全力保障九大集团公司正常运转；进一步完善出资人制度，健全企业薪酬分配制度，开展市属国有企业分类监管，促进国资国企降本增效。深化投融资体制改革，加快建立多元化的投融资机制，引导社会资本扩大投资。推进预算管理制度改革，建立财政资金统筹管理机制，加强政府债务限额管理。积极稳妥发展混合所有制经济，完善非公有制经济发展政策，激发民间资本活力。

　　深化行政审批制度改革。深入推进“八个一”综合改革，继续减少、下放行政审批事项，调整完善市本级政府部门权责清单。发展“互联网+政务服务”模式，健全全市统一的公共资源交易平台，完善公共资源交易规则，实行公共资源交易目录化、动态化管理。全面落实不动产统一登记制度，完善不动产登记信息平台，缩短办结时限和抵押登记时限。发挥企业信用信息公示系统作用，构建以信用监管为主的事中事后监管机制。深入推进商事制度改革，巩固和扩大“六证合一、一照一码”改革成果。推动综合行政执法体制改革，更好地为企业、为市民服务。

　　统筹实施社会领域改革。实施农村综合改革，稳步推进供销社、国有林场改革发展，推动农村土地规范有序流转，年内基本完成农村土地承包经营权确权登记颁证工作。深化户籍制度改革，完善公共服务、社会保障制度，有序推进农村转移人口市民化。实施国税、地税征管体制改革，加快现代化税收征管体制和征管能力建设。统筹文化、教育、医药卫生、社会保障、食品药品安全、安全生产监管体制等领域改革，不断健全发展体制机制。

　　七、全面扩大开放与合作，不断增强经济发展动力活力

　　提高开放型经济发展水平。主动参与国家“一带一路”、珠江—西江经济带建设，借助中国—东盟博览会等平台资源，办好中国—东盟汽车工业博览会。开展立足西南中南、面向港澳与东盟的多领域交流合作，推动与广佛肇经济圈的产业对接，促进跨区域各产业各领域的交流合作。建立更加务实的“柳来河”工作机制和磋商机制，促进“柳来河”三地产业互动、交通互联互通。实施新一轮“加工贸易倍增计划”，鼓励和支持汽车、工程机械、钢铁等传统优势产业走出去。建设粤桂黔高铁经济带合作试验区广西园、中欧战略合作示范园，加快柳州保税物流中心（B型）申报，规划建设空港经济区，努力构建全方位开放合作的新格局。

　　拓展对外交通发展网络。加快建设柳州站站房扩建工程（东站房），开工建设柳州动车存放所，实施焦柳铁路怀化至柳州段电气化改造、湘桂铁路柳州枢纽扩能工程，加快柳州至广州、柳州至韶关、柳州至河池等铁路项目前期工作。着力构建高效便捷公路网络，开工建设柳州经合山至南宁、融水至河池等高速公路，加快柳州至南宁改扩建、柳州至梧州等高速公路建设，确保三江至桂林等高速公路建成通车。推进柳江红花二线船闸、柳州港官塘作业区一期工程、鹿寨江口作业区建设，进一步提升港口吞吐能力。加大柳州机场航线开发力度，积极申报柳州白莲机场临时航空口岸，开展机场远距离二跑道前期研究工作。

　　狠抓招商引资工作。加大对战略性新兴产业、汽车全产业链、智慧电网产业、轨道交通产业链、建筑产业现代化等领域招商引资工作，一个项目一个团队推进项目落地建设，营造重商、亲商、安商、护商的良好环境。年内新引进超亿元项目100个以上、超十亿元项目15个以上，实现全年市外到位资金目标任务增长7%以上、实际利用外资（全口径）目标任务增长10%。

　　八、增强城市综合服务功能，营造山清水秀的自然生态

　　进一步完善城市基础设施。推进新一轮柳州市城市总体规划编制工作，强化控制性详细规划的管控。编制柳州城市色彩规划，着力构建层次分明、整体优化、搭配合理、比例协调的城市色彩体系。加快轨道交通申报工作，启动建设轨道交通1号线，开工建设轨道交通三门江跨江大桥。建设柳东新区国家产城融合示范区，实施雒容至东泉公路项目，延伸柳东新区发展空间。将柳江区规划、建设纳入市级管理，实施柳江城市建设十大工程。全面建设北部生态新区，以生态为核心科学编制发展规划、建设规划、产业规划以及各专项规划，打造一个以城带乡、城乡互动、产城融合、生态绿色的新型城镇化建设示范新区和智慧城市新区。加强历史街区保护整治，继续实施旧城、城中村、棚户区改造。开展海绵城市试点，推进莲花山片区“慢城”建设，实施地下综合管廊项目，全面完成市区内涝积水点整治。建设 “智慧城市”，打造智慧柳州时空信息云平台。优先发展公共交通，着力解决城市拥堵问题，促进城市管理智能化、网络化、科学化。

　　建设绿色低碳的生态市。积极创建国家生态市、国家生态园林城市，推进花园城市2.0版建设，实施三门江国家森林公园保护利用提升工程，建设洋紫荆主题公园、北部生态新区湿地公园、官塘滨江湿地公园和柳江岜公塘湿地公园。进一步强化环保责任追究，严控主要污染物排放。全面推行河长制，深入开展柳江河重点流域、水源保护区综合治理，确保柳江河饮用水保护河段保持国家地表水Ⅲ类以上水质标准。大力发展生态经济，建设国家循环经济示范市，力争成为国家第三批低碳城市试点。依托现有城市生活垃圾处理场所，规划建设以生活垃圾、建筑垃圾、医疗废弃物、污泥及其他废弃物资源化利用为主的静脉产业园。启动“美丽柳州·宜居乡村”建设，实施“产业富民、服务惠民、基础便民”专项活动，集中资金、资源打造“宜居乡村”综合示范村屯。

　　九、持续保障和改善民生，营造团结和谐的社会生态

　　提升民生保障水平。大力推动“大众创业、万众创新”，进一步鼓励人才创新，畅通人才合理流动渠道，加快引进和培养高层次创业创新人才。统筹推进高校毕业生、农村转移劳动力、城镇就业困难人员等重点群体就业创业。不断增强社会保障能力，全面启动社会保险标准化建设“先行城市”活动，完成城镇居民医保和新农合的整合，建立统一的城乡居民基本医疗保险制度，简化城镇医疗保险报销程序，提高城乡最低生活保障标准。开展政府购买居家养老服务扩标提面工作，推进休闲旅游养生养老产业发展，着力构建“两环一中心”养老产业发展布局。大力推进民生保障工程，完成1万套保障性住房改造及新建任务。

　　协调发展社会各项事业。从群众关注度高的问题入手，着力推动公共服务公平均衡，增强人民群众的获得感。

　　——优先发展教育事业。健全学区制管理、集团化办学工作机制，完善教师轮岗交流、县属轮教配套政策，优化城乡教育资源。启动第三期学前教育三年行动计划，力争学前教育三年毛入园率达82%。大力推进义务教育均衡发展，加快义务教育学校标准化建设，提高保障水平。改善高中阶段教育学校办学条件，推进一批示范、特色普通高中建设，提高学生综合素质。完善特殊教育体系，提高适龄“三残”少年儿童入学率。做优职业教育，加快建设广西（柳州）公共实训基地，推进“全国现代学徒制试点城市” “广西现代职业教育改革发展示范区”建设，促进学历教育向职业资格准入转变。利用高等院校引进智能制造领军人才、创新团队、高端人才，加快广西科技大学鹿山学院向应用型本科院校转型升级，筹建柳州理工学院。

　　——繁荣发展文化体育事业。大力发展文艺事业，提升文化惠民项目品牌，支持《侗》《八桂大歌》等艺术精品“走出去”。创建第三批国家公共文化服务体系示范项目，打造新的文化惠民平台，丰富群众文化活动。做好文化遗产保护利用。推动文化产业转型升级，办好水上狂欢节、世界水上极速运动大赛、柳州世界铁人三项赛等重大赛事活动。促进全民健身与全民健康深度融合，广泛开展群众体育健身活动。积极争创全国文明城市，深入开展社会主义核心价值观教育，广泛开展精神文明、民族团结创建活动，弘扬柳州精神。

　　——建设健康柳州。积极推进健康城市建设，做好国家卫生城市复审工作，提高医疗救助、卫生监督、疾病防控、妇幼保健工作水平。深化城市公立医院改革，扩大现代医院管理制度试点，探索建立公立医院绩效考评机制。推进紧密型区域医疗联合体建设，完善分级诊疗体系。促进中医药发展，加强基层医疗卫生服务能力建设，促进基本公共卫生服务均等化。实施全面两孩政策和优生优育工程，不断优化人口结构。深入创建“食品安全城市”，加大食品药品监管力度，严厉打击食品药品违法犯罪行为，保障人民群众饮食用药安全。

　　——提升社会治理水平。建设“平安柳州”，深入推进社会治理现代化建设，打造以智慧公安、智慧司法为主要构架的社会治理信息化管理平台。全面加强社区建设，新建、购置和改扩建101个城市社区服务场所，推进社区网格化管理，“零距离”为居民做好服务。加快完善社会信用体系，建设诚信柳州。深入实施“领导干部包案”制度，做好矛盾纠纷化解。加强法治宣传教育，提高全社会尊法学法守法意识。坚决落实安全生产责任制，切实做到“党政同责、一岗双责、齐抓共管、失职追责”。抓好信访应急维稳工作，全力维护社会和谐稳定。

　　——推进军民融合深度发展。持续深化拓展全民国防教育，积极支持军队改革，注重在经济建设中贯彻国防需求，稳步推进国防动员准备和后备力量建设改革，开展好征兵工作，切实做好军事设施保护。加大双拥创建力度，加强基础设施等重要领域融合发展，形成军民深度融合的发展格局。

　　十、建设法治政府、廉洁政府和服务型政府，营造风清气正的政治生态

　　推进法治政府建设。坚持依法行政，履行党政主要负责人推进法治建设第一责任人职责。完善政府立法工作机制，科学制定和开展重点领域政府立法。建立健全综合执法制度，开展食品药品、工商质监、公共卫生、农林水利、交通运输等领域综合执法，推动综合行政执法工作向乡镇延伸。进一步完善行政执法程序，规范执法行为，营造公平公正的社会环境。

　　完善政府决策机制。严格执行《广西壮族自治区重大行政决策程序规定》，健全重大行政决策工作机制。大力推行政务公开、政府信息公开，实行重大决策预公开制度，落实重大决策社会稳定风险评估，健全公职律师管理制度。自觉接受市人大及其常委会的法律监督和工作监督，主动接受市政协的民主监督，认真办理人大议案、建议和政协提案。广泛听取民主党派、工商联、无党派人士意见，支持工会、共青团、妇联等人民团体发挥作用。

　　进一步强化作风建设。巩固“两学一做”学习教育成果，全面贯彻落实中央“八项规定”精神和国务院“约法三章”。认真履行党风廉政建设“一岗双责”，严肃查处各类违纪违法案件。开展“不作为、乱作为”专项整治，坚决杜绝“议而不决、决而不施、施而无效”的行为。严格控制“三公”经费。加大对政策措施落实、公共财政资金、政府投资项目等审计力度，健全决策部署、重大项目推进落实的督查机制。完善绩效考评体系，发挥绩效激励导向作用。继续开展“市长接待日”“市长企业接待日”“中小企业服务月”和领导公开大接访活动，密切与群众的血肉联系，树立为民、务实、清廉的政府形象。

　　各位代表，“空谈误国，实干兴邦”。今年柳州发展的目标任务已明，关键在于抓落实。让我们紧密团结在以习近平同志为核心的党中央周围，在自治区党委、政府和市委的坚强领导下，切实把思想和行动统一到中共柳州市第十二届代表大会第二次会议确定的目标任务和工作要求上来，坚定信心，主动作为，真抓实干，加快实现“率先建成”和“打造龙头”双目标，以优异成绩迎接党的十九大胜利召开！
